# Correction: The Return Trip Is Felt Shorter Only Postdictively: A Psychophysiological Study of the Return Trip Effect

**DOI:** 10.1371/journal.pone.0133339

**Published:** 2015-07-15

**Authors:** Ryosuke Ozawa, Keisuke Fujii, Motoki Kouzaki

## Notice of Republication

This article was republished on June 27, 2015, to correct an error in the title. The title erroneously said "The Return Trip Is Felt Longer" instead of "The Return Trip Is Felt Shorter." Please download this article again to view the correct version. The originally published, uncorrected article and the republished, corrected article are provided here for reference.

## Supporting Information

S1 File Originally published, uncorrected article. (PDF)

S2 File Republished, corrected article. (PDF)
