# Supplementary material for: Correction: The Return Trip Is Felt Shorter Only Postdictively: A Psychophysiological Study of the Return Trip Effect
Source: PLoS One. 2015 Jul 15;10(7):e0133339. doi: 10.1371/journal.pone.0133339 (PMC4503449; doi:10.1371/journal.pone.0133339)
Supplement: S2 File — (PDF) [file pone.0133339.s002.pdf]

PLOS | ONE

RESEARCH ARTICLE

The Return Trip Is Felt Shorter Only Postdictively: A Psychophysiological Study of the Return Trip Effect

Ryosule Ozawa[1¤a¤b], **Keisuke Fujii**[1,2¤b¤c], **Motoki Kouzaki**[1]*

1 Laboratory of Neurophysiology, Graduate School of Human and Environmental Studies, Kyoto University, Kyoto, Japan, **2** Japan Society for the Promotion of Science, Tokyo, Japan

¤a Current Address: Dynamic Brain Network Laboratory, Graduate School of Frontier Biosciences, Osaka University, Osaka, Japan
¤b Current Address: Japan Society for the Promotion of Science, Tokyo, Japan
¤c Current Address: Research Center of Health Physical Fitness and Sports, Nagoya University, Aichi, Japan
* kouzaki.motoki.4x@kyoto-u.ac.jp

CrossMark
click for updates

OPEN ACCESS

Citation: Ozawa R, Fujii K, Kouzaki M (2015) The Return Trip Is Felt Shorter Only Postdictively: A Psychophysiological Study of the Return Trip Effect. PLoS ONE 10(6): e0127779. doi:10.1371/journal.pone.0127779

Academic Editor: William J. Matthews, University of Cambridge, UNITED KINGDOM

Received: June 27, 2014

Accepted: April 19, 2015

Published: June 10, 2015

Copyright: © 2015 Ozawa et al. This is an open access article distributed under the terms of the Creative Commons Attribution License, which permits unrestricted use, distribution, and reproduction in any medium, provided the original author and source are credited.

Data Availability Statement: All relevant data are within the paper.

Funding: These authors have no support or funding to report.

Competing Interests: The authors have declared that no competing interests exist.

Abstract

The return trip often seems shorter than the outward trip even when the distance and actual time are identical. To date, studies on the return trip effect have failed to confirm its existence in a situation that is ecologically valid in terms of environment and duration. In addition, physiological influences as part of fundamental timing mechanisms in daily activities have not been investigated in the time perception literature. The present study compared round-trip and non-round-trip conditions in an ecological situation. Time estimation in real time and postdictive estimation were used to clarify the situations where the return trip effect occurs. Autonomic nervous system activity was evaluated from the electrocardiogram using the Lorenz plot to demonstrate the relationship between time perception and physiological indices. The results suggest that the return trip effect is caused only postdictively. Electrocardiographic analysis revealed that the two experimental conditions induced different responses in the autonomic nervous system, particularly in sympathetic nervous function, and that parasympathetic function correlated with postdictive timing. To account for the main findings, the discrepancy between the two time estimates is discussed in the light of timing strategies, i.e., prospective and retrospective timing, which reflect different emphasis on attention and memory processes. Also each timing method, i.e., the verbal estimation, production or comparative judgment, has different characteristics such as the quantification of duration in time units or knowledge of the target duration, which may be responsible for the discrepancy. The relationship between postdictive time estimation and the parasympathetic nervous system is also discussed.

Introduction

Our perception of time is a guiding force in our behaviors because it is an essential component of cognition and motor performance, representing one of the basic mechanisms of cerebral function [1]. To deal with time, multiple systems over more than ten orders of magnitude have been developed because we process and use temporal information across a wide range of intervals [2]. Time perception researchers often separate time into millisecond timing, interval timing including the range of seconds-to-minutes-to-hours, and circadian timing [2]. In this paper we call timing in the range of minutes-to-hours "real-life" timing in order to highlight its relevance to our daily life. Interval timing is less accurate than other timing ranges [2,3]. Because of this inaccuracy, we experience many odd phenomena related to time perception. For example, when we go from a station to a destination, and return to the same station, the return trip often seems shorter than the outward trip, though the distance traveled and the actual duration of the trips are almost identical. This phenomenon is called the "return trip effect" [4].

Zakay [5] discussed this effect from the viewpoint of time relevance, which indicates how important it is in a specific situation to be aware of the passage of time. The higher the time relevance, the more attentional resources will be allocated to time and therefore the longer the estimate of duration. When we have to go somewhere at a certain time for an important event, time relevance is high. On the contrary, when returning to the starting point, time is not so important and time relevance is low. However, two studies directly examining the return trip effect provide other potential explanations. These studies did not include a purpose for the outward trip; therefore, time relevance seemed to be equal between outward and return trips. Ven et al. [4] confirmed that the return trip effect is frequently experienced in daily life. They also reported that it is not due to an increase in familiarity with a route, but is probably due to a violation of expectations for the durations of trips: the more the participants' expectations were violated on the initial trip, the more they experienced the return trip effect. Seno et al. [6] conducted a virtual travel experiment with verbal instructions and examined two factors: one perceptual (optic flow inducing self-motion perception or random dot control condition) and one cognitive (with or without a round trip story). Their results indicate that the return trip effect is induced only when self-motion perception is accompanied by the round-trip story, in other words, by combined perceptual and cognitive factors.

The foregoing studies provide important suggestions about the return trip effect, but there are also some problems. One is that a comparison between the round-trip condition and non-round-trip condition in an environment close to daily experience is needed. Ven et al. [4] used actual trips, or virtual trips by movies, but they compared only round-trip conditions, without a control condition. Seno et al. [6] examined the round-trip and non-round-trip conditions, but their experimental environment seems to be far from actuality, and the duration of the task (40 s) was much shorter than real-life trips. Recently, the need for ecologically valid tasks has been discussed [7–9]. To address these issues, we investigated not only the round-trip condition but also the non-round-trip condition by presenting walking movies for relatively long intervals. The duration of a trip in this study was over 20 min, which is closer to typical trip-durations than previous studies. The experimental setup using walking movies is more ecological than that in Seno et al. [6] and the same as that in Ven et al. [4]. In one of our unpublished studies, when participants walked on a treadmill during the same experiment setup, they sometimes tried to turn right or left on the treadmill as if they had walked in a real environment. The method of watching a movie presented by a projector in a dimly room seems to have a sufficient sense of immersion, though we acknowledge that watching a movie is different from a real walk. From the viewpoint of duration interval and environment, this study is comparatively ecologically valid.

A second issue is the need for prospective timing for a long real-life interval. Time perception studies are divided into prospective and retrospective timing [1,10,11]. Prospective timing is involved in the situation where participants are alerted in advance that timing is an essential part of the task presented, for instance, you are asked to perform arithmetic exercises for a given duration and asked in advance to estimate the duration upon the completion of the interval. This timing depends on attentional processes, as explained by the attentional gate model [5,7–9,12,13]: the attention paid to the duration closes a switch between an intrinsic pacemaker and a pulse accumulator, and time judgment is based on the pulses counted in the accumulator. As a result, the more attention is paid to the duration, the longer time is felt to be. Retrospective timing is the situation where participants are asked an unexpected question about duration, for example, you try to recall how long a film was, or how long it took to talk with friends. Retrospective timing is based on memory processes [5,7,9,12,13], and a larger memory for an event leads to a longer remembered duration. When estimating time, it has been assumed that the amount of segmentation determines the size of a memory as a contextual change model indicates [14,15]: the contextual changes perceived generate temporal referents in memory and we reconstruct the duration of the event based on them. That is, more mental contextual segmentations lead to longer estimation. Ven et al. [4] used the retrospective paradigm. On the contrary, Seno et al. [6] used the prospective paradigm, but as mentioned above the duration of the task was very short. Therefore, it is unclear whether the return trip effect is observed in prospective timing for longer, real-life intervals. We adopted two methods of time estimation. One was repeated production of a 3 min interval (RP3), which reflects time perception in real time, or prospective timing. The other method was an 11-point scale reflecting postdictive time perception, or retrospective timing, as it was also used in a previous study [4]. Using RP3 and an 11-point scale enabled us to evaluate both prospective and retrospective timings within the same experiment. However, it should be noted that we use the terms "time perception in real time" and "postdictive time perception."

It is important that the return trip effect has been observed when using the verbal estimation method [4,6] and the comparison method [4]. The estimation method may be a more complex time judgment, because it implies the quantification of duration in time units while the comparison method only requires a comparison between durations [8]. Regardless of this difference the return trip effect has occurred. In this study, RP3 as the production method and an 11-point scale as the comparison method were used. The production method is compatible with the verbal estimation method [1]. Based on the observations in previous studies, we hypothesized that the return trip effect would be observed not only in the postdictive rating task but also in RP3.

Studies of time perception have focused on physiological factors such as heart rate (HR), body temperature, or age, as well as perceptual or cognitive factors, in search of fundamental timing mechanisms [1,10,16]. Classically the relationship between time perception and body temperature has been well known. The general rationale is that, as increase in temperature facilitates chemical reactions, any physiologically based pulser or oscillator will operate at a faster rate, with decrease in temperature having the opposite effect [10]. Compared to body temperature, HR may have more complex effects. Jamin et al. [17] found a linear relationship between time estimation and HR, with underestimation of duration with decreased HR. This seems to be explained by the same rationale as that for body temperature because a decrease in HR may lead to a slower rate of the physiologically based pulser, which can cause underestimation of duration. Lediett & Tong [18] indicated that increases in HR improved the accuracy of time perception in some participants, but lessened it in other participants, depending on their personality. Though the direction of the effect of HR is unclear, HR can modulate time perception. Moreover, HR can be analyzed in more detail. HR is regulated by the sympathetic and

parasympathetic nervous systems; therefore, HR variability (HRV) represented by the standard deviation (SD) includes the influence of both systems [19]. Analyses such as spectral analysis or the Lorenz plot can separately evaluate these modes of regulation [20–23]. Measurement of HR enables us to use these analyses, which is the advantage over measurement of body temperature.

While these physiological factors that are assumed to underlie timing mechanisms are mainly investigated over relatively short intervals, perception for long intervals is attributed to cognitive processes such as memory or attention. However, it is not denied that physiological factors may also affect time perception for long intervals. HR and HRV seem to be related to cognitive processes as well as autonomic regulation. HR has been found to react to the emotional valences of film clip stimuli while HRV has been found to be related to acoustic startle reflex sensitive to negative stimuli [24]. It is possible that these physiological responses could not only underlie the oscillator of the internal clock but also modify time perception for long intervals through more complex cognitive processes such as emotion [13,25].

The aims of this study were 1) to compare the round-trip and non-round-trip conditions with a real-life duration and comparatively ecological environment, 2) to identify the circumstances where the return trip effect occurs (i.e., time perception measured in real time or postdictively), and 3) to examine whether autonomic nervous system (ANS) activity contributes to the return trip effect. We hypothesized that the return trip effect would be observed in both RP3 and the 11-point scale, and that differences in ANS activities between the two groups may underlie the return trip effect.

Materials and Methods

Participants

Twenty healthy males (aged 20–30 years) participated in the study. All participants reported normal or corrected-to-normal vision. The experimental procedures were conducted in accordance with the Declaration of Helsinki and were approved by the Local Ethics Committee of the Graduate School of Human and Environmental Studies, Kyoto University. Participants gave written informed consent according to institutional guidelines.

Procedure and tasks

The experiment consisted of two test sessions: the first trip session and the second trip session. In both sessions, participants were asked to watch a movie recorded while walking. Before each session, they were handed a map of a route they would watch in the movie and instructed to glance at the map during the task as if they actually walked the route for the first time. There were three different movies: movie-1, -2, and -3 (Fig 1). Movie-1 showed a route from "S" to "E" in Fig 1A. Movie-2 showed a route from "(S)" to "(E)" in Fig 1A, which meant that the route was the same as that of movie-1, but the direction of travel was reversed. Movie-3 showed a route from "S" to "E" in Fig 1B, which was completely different from those of movie-1 and movie-2. The durations and distances of the three movies were equal (26.3 min, 1.7 km). A round-trip group, comprising 10 participants, watched movie-1 or movie-2 in each session. A control group, comprising the other 10 participants, watched movie-2 or movie-3 in each session. The order of movies was counterbalanced across participants in both groups. We confirmed that all twenty participants were unfamiliar with the routes they had watched.

While watching the movie, participants were required to verbally report when they felt it had taken 3 min, and to continue these reports until the end of the movie (repeated production of a 3 min interval task hereafter called RP3 task). After watching the two movies, they were asked which movie they felt was longer on an 11-point scale from −5 (the first was a lot longer)

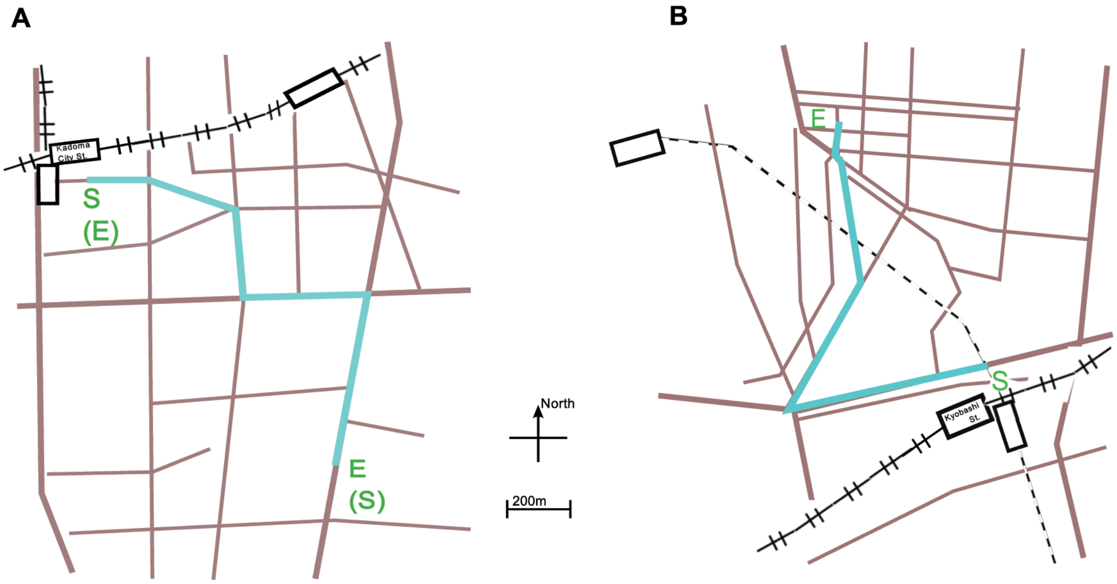

Fig 1. Maps of routes displayed in movies. 'S' on the maps denotes the starting point, and 'E' the endpoint for each route. Cyan line represents the routes of movies. (A) A route in movie-1 and -2, with 'S' and 'E' for movie-1 and 'S' and 'E' with parentheses for movie-2. (B) A route in movie-3.

doi:10.1371/journal.pone.0127779.g001

to +5 (the second was a lot longer). They were not informed of this question in advance. Participants were instructed to remove their wristwatch or any rhythmical devices and not to use verbal nor nonverbal counting strategies such as "1, 2, 3. . ." during the tasks. Before the experimental sessions, there was a practice session in which participants watched a movie, saw a map, and carried out the RP3 task using a route that was different from those used in the test sessions. There was a rest interval of 10 min between sessions.

Apparatus

The experimenter had recorded four movies (movie-1, -2, -3, and the movie used in the practice session) using a camera (EX-F1, HD/30 fps, CASIO, Tokyo) held in front of the chest while walking. We carefully prepared three experimental movies to precisely match their durations. Firstly, an experimenter who would record the movies practiced walking in order to walk with constant speed. Secondly, we preliminarily searched routes to examine the timings when traffic lights change so that we could adjust the frequency of being stopped by red traffic lights. Finally, we shot each movie four to six times. Based on these efforts, we produced movies with well-controlled durations. The movies used in the first and second test sessions were approximately 26.3 min long, and the movie used in the practice session was approximately 9.0 min long. Movies were played back by a PC and presented on a screen by a projector (NP62, NEC, Tokyo) at a size of 0.9 m × 1.5 m. Participants were individually tested in a dimly lit room and comfortably sat on a chair. The distance between the screen and the projector was approximately 2.70 m, and that between the screen and the chair was approximately 3.65 m. At the start of the movie, a stopwatch was started, and the experimenter filmed the session so that the times of participants' verbal reports could subsequently be confirmed. To obtain heart beats, a bipolar electrocardiogram (ECG) was continuously measured by a precordial lead. The recorded ECG was stored on a computer via 16-bit analog-digital converter (PowerLab 16SP, ADInstrument, Sydney) at a sampling frequency of 1 kHz.

Data and analyses

Two indices were used to evaluate time perception. RP3 represented the objective durations between the start of the movie and the first report, or between a report and the following report produced by participants in the RP3 task. The larger the RP3, the shorter the participant evaluated the past time was because overproduction in the production method equals to underestimation in the verbal estimation method. This index evaluated time perception in real time because it was produced during the experiment. The other index was the 11-point scale. This index of time perception more closely corresponds with our daily experiences. Also the judgment on the 11-point scale was not processed during the tasks because it was unexpectedly asked in the end. Therefore this judgment was constructed after the tasks.

ANS activity was assessed from the ECG data. Detection of each cardiac impulse was triggered by the R wave, and visual inspection was used to search the possibility of extra or missing beats. Then R-R intervals were calculated from these impulses, and were converted into instantaneous HRs. To investigate overall changes in HR, the mean instantaneous HR and the SD of instantaneous heart rates (SD-HR) were calculated. SD-HR is considered to be an index reflecting the activity of the whole ANS, because the SD of HR reflects all cyclic components responsible for variability, and the variance is mathematically equal to the total power in spectral analysis [19]. To investigate ANS activity in detail, the Lorenz plot was adapted. This is a two-dimensional non-linear plot. When the sequence of the consecutive R-R intervals is expressed by $I_1, I_2, \ldots, I_n$, the Lorenz plot is constructed by plotting I_{k+1} against I_k. Two components of the R-R fluctuation are calculated from the plots: the length of the transverse axis (T), which is vertical to the line $I_k = I_{k+1}$, and that of the longitudinal axis (L), which is parallel with the line $I_k = I_{k+1}$. These components are calculated by quadrupling the SDs of the plotted points along its axis. Two autonomic indices were obtained from these components: cardiac vagal index (CVI) is defined as $\log_{10}(L \times T)$ and cardiac sympathetic index (CSI) as L/T. CVI and CSI reflect parasympathetic and sympathetic functions, respectively. This analysis is more sensitive than spectral analysis [20].

RP3s were averaged within participants in each session. ECG data were separated into segments corresponding to RP3s. Then HR, SD-HR, CVI, and CSI were calculated in each segment and averaged across segments within participants in each session.

Statistics

To assess the independent and combined effects of RP3, HR, SD-HR, CVI, and CSI, a two-way mixed-model analysis of variance (AVOVA) was conducted with the round-trip and control groups as a between-subjects factor (Group) and the first and second trips as a within-subjects factor (Trip Session). If a significant interaction was found, within-subjects differences were analyzed for each group using two-tailed pair-wise *t* tests. To assess the 11-point scale, a two-tailed Welch's *t* test was used because of the difference of variance mentioned in Results (see also Fig 2B). Also, a two-tailed one-sample *t* test was used for each group to judge whether the estimation was significantly biased. Effect size was estimated by using partial eta-squared (η_p^2) and Cohen's *d*. Pearson correlations between autonomic nervous activities (the change of HR, SD-HR, CVI, and CSI) and time estimates (changes in RP3, and the values of 11-point scale) were investigated in each group. The change in each index was defined by subtracting the value in the second trip session from that in the first trip session. For all statistical calculations, $p <. 05$ was accepted as significant. In case of multiple comparisons at follow-up analyses, Holm correction was used to control for false positives.

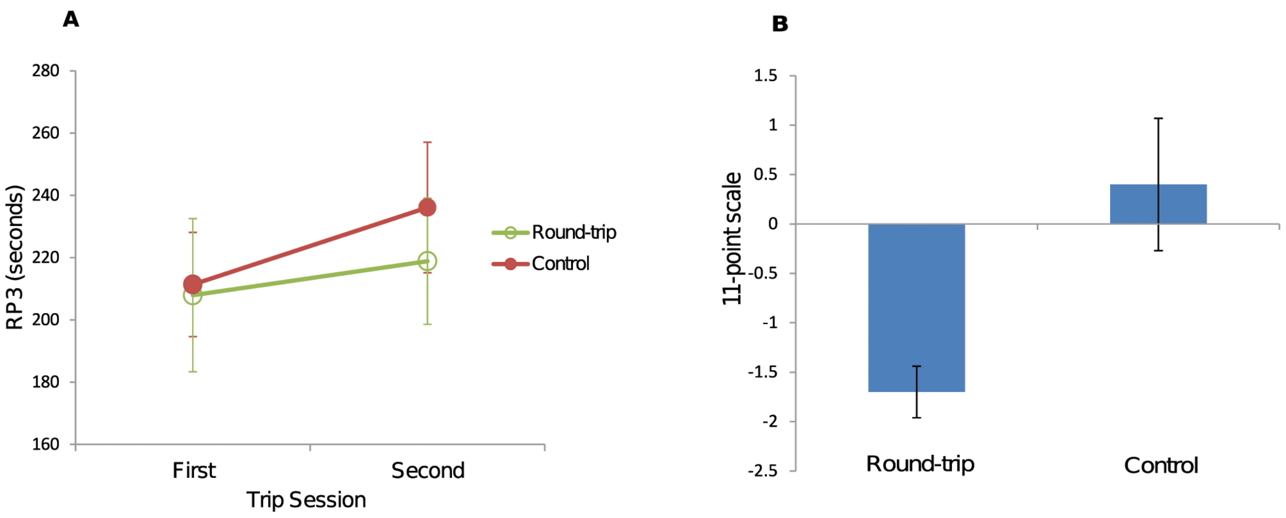

Fig 2. Time estimations. (A) Mean RP3 in each condition, calculated across participants, and (B) mean 11-point scale in each group, calculated across participants. Values are means ± 1SE. RP3, repeated production of a 3 min interval.

doi:10.1371/journal.pone.0127779.g002

Results

Time estimation

The mean RP3s are plotted in Fig 2A. An ANOVA on RP3 revealed that there was a significant effect of Trip Session ($F(1, 18) = 5.57, p = .03; \eta_p^2 = .24$). There was no effect of Group ($F(1, 18) = .13, p = .72; \eta_p^2 = .007$) and no significant Trip Session × Group interaction ($F(1, 18) = .84, p = .37; \eta_p^2 = .04$).

The mean 11-point scale scores are plotted in Fig 2B. There was an apparent difference in SE between two groups. In the round-trip group the evaluated scores were all negative whereas in the control group the scores included both negative and positive values. Due to this difference we performed a Welch's *t* test showing that there was a significant difference between the two groups ($t(12) = -2.92, p = .013; d = -1.31$). A one-sample *t* test showed that the mean score for the round-trip group was smaller than 0 ($t(9) = -6.53, p = 1.1 \times 10^{-4}; d = -2.06$). In addition, all ten participants produced negative values. The scores on the 11-point scale for the control group did not differ from 0 ($t(9) = .60, p = .57, d = .19$).

Autonomic nervous function

Variables related to ANS activities are plotted in Fig 3. In the round-trip group one participant showed very slow HR (around 55 beats/min) with low variability because he was a skilled sport player, and another showed very fast HR (around 100 beats/min) with high variability, which led to wide distributions of HR and SD-HR within the group (Fig 3A and 3B). An ANOVA on HR (Fig 3A) revealed that there was no effect of Trip Session ($F(1, 18) = .10, p = .75; \eta_p^2 = .006$) or Group ($F(1, 18) = .14, p = .71; \eta_p^2 = .008$), and no interaction ($F(1, 18) = .70, p = .42; \eta_p^2 = .04$). On SD-HR (Fig 3B), there was no effect of Trip Session ($F(1, 18) = 1.77, p = .20; \eta_p^2 = .09$) or Group ($F(1, 18) = .77, p = .39; \eta_p^2 = .04$), but there was a significant Trip Session × Group interaction ($F(1, 18) = 5.16, p = .036; \eta_p^2 = .22$). Two-tailed pair-wise *t* tests revealed that SD-HR in the second trip session was larger than that in the first trip session for the control group ($t(9) = -3.40, p = .016; d = -.48$), and that there was no difference between trip sessions for the round-trip group ($t(9) = .55, p = .59; d = .08$). On CVI (Fig 3C), there was no effect of Trip Session ($F(1, 18) = .51, p = .48; \eta_p^2 = .03$) or Group ($F(1, 18) = 2.30, p = .15; \eta_p^2 = .11$), and no

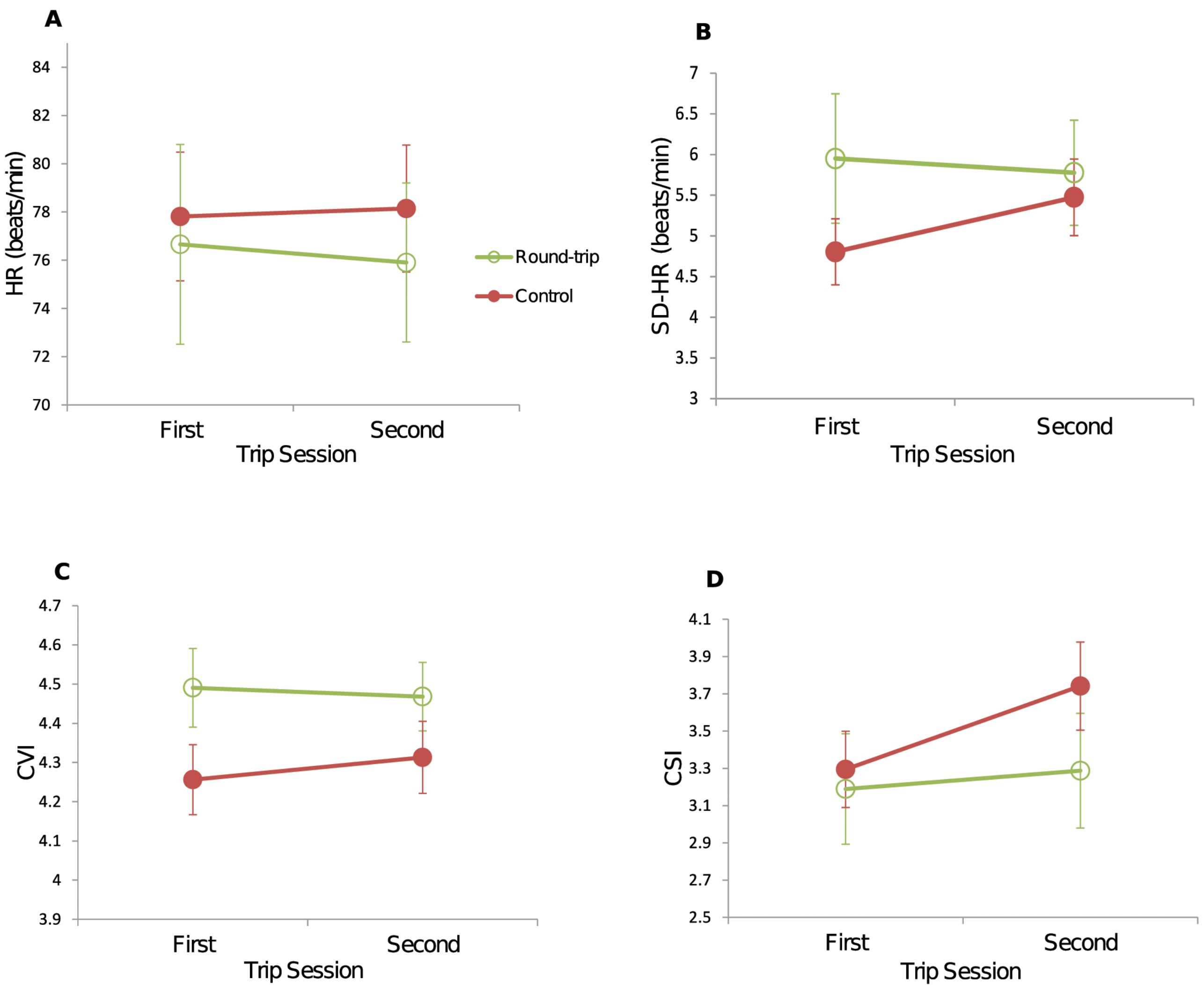

Fig 3. Physiological indices. (A) Mean HR in each condition, (B) mean SD-HR in each condition, (C) mean CVI in each condition and (D) mean CSI in each condition, calculated across participants. Values are means ± 1SE. HR, heart rate; SD-HR, standard deviation of heart rate; CVI, cardiac vagal index; CSI, cardiac sympathetic index.

doi:10.1371/journal.pone.0127779.g003

interaction ($F(1, 18) = 2.74$, $p = .12$; $\eta_p^2 = .13$). On CSI (Fig 3D), there was a significant effect of Trip Session ($F(1, 18) = 9.47$, $p = .006$; $\eta_p^2 = .35$), but no effect of Group ($F(1, 18) = .59$, $p = .45$; $\eta_p^2 = .03$). The Trip Session × Group interaction approached significance ($F(1, 18) = 3.87$, $p = .065$; $\eta_p^2 = .18$). This interaction was not significant, but *t* tests showed that CSI in the second trip was larger than that in the first trip session for the control group ($t(9) = -4.130$, $p = .005$; $d = -.64$), and that there was no difference between trip sessions for the round-trip group ($t(9) = -.70$, $p = .50$; $d = -.10$).

Correlations

Correlations between ANS activities and time estimates are presented in Table 1. A significant correlation between the 11-point scale and the change in CVI was found in the control group ($r = .74$, $p = .014$) (Fig 4A). The correlation between the 11-point scale and the change in SD-HR approached significance in the control group ($r = .62$, $p = .054$) (Fig 4B). No other

Table 1. Correlation coefficients between ANS and time perception for the round-trip group and the control group.

	Round-trip				Control			
	RP3		11-point scale		RP3		11-point scale	
	r	*p*	*r*	*p*	*r*	*p*	*r*	*p*
HR	-.49	.149	-.45	.197	-.32	.370	-.41	.240
SD-HR	-.26	.474	-.36	.314	.08	.828	.62	.054
CVI	.23	.532	-.07	.853	.33	.347	.74	.014*
CSI	-.29	.416	-.37	.287	-.16	.656	.35	.316

* p <. 05.

doi:10.1371/journal.pone.0127779.t001

significant correlation was found in the control group, and no correlations were found in the round-trip group.

Discussion

It should be noted that we cannot be confident of the null results of ANOVA interaction effects mentioned below due to low statistical power. Assuming sample size = 10 per group and medium effect size $f = .25$ equivalent to $\eta_p^2 = .06$, the statistical power to detect the interaction = .56 (calculated with G*Power 3.1 [26]).

Discrepancy between the two time estimates

We assessed time perception in two ways, prospective judgment involving on-going temporal production (RP3) and retrospective judgment comprising a comparison of two intervals on an 11-point scale. These two indices apparently showed different results, suggesting that the return trip effect might be caused only postdictively. According to the 11-point scale results, only the round-trip group estimated that the second trip took less time than the first trip. In contrast, the RP3 results indicate that both groups felt that time had been shorter in the second trip session. Considering that the 11-point scale is a similar method for evaluating time perception to that used in previous research [4] and is also close to the situation in which we

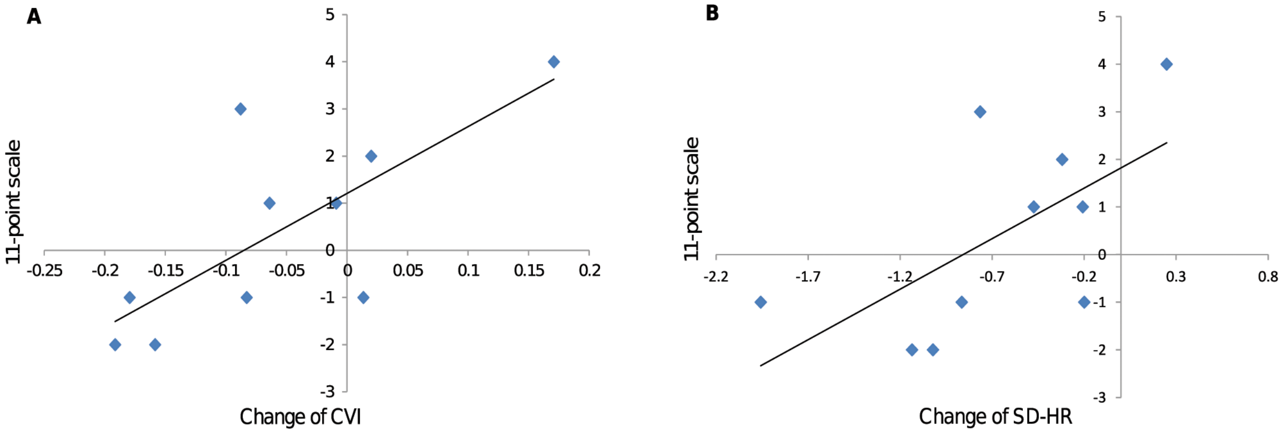

Fig 4. Relations between ANS and postdictive time perception. (A) Correlation of the change of CVI with 11-point scale for the control group, and (B) correlation of the change of SD-HR with 11-point scale for the control group. The change was defined as subtracting the value in the second trip session from that in the first trip session. ANS, autonomic nervous system; CVI, cardiac vagal index; SD-HR, standard deviation of heart rate.

doi:10.1371/journal.pone.0127779.g004

experience the return trip effect in daily life, it is certain that the return trip effect is observed at least postdictively. In addition, this difference between the two time estimates suggests that postdictive time perception, measured by the 11-point scale, might not be based on time perception in real time, as estimated by RP3. During tasks, time may be felt to be shorter in the second trip session for both groups, but this would not lead to the same experience of time after completion of the tasks.

The discrepancy between the two estimates can be explained by timing strategies. Time perception includes prospective and retrospective timing [1,10,11]. RP3 would be prospective timing because participants were aware of this task during the experiment, and the production method is a major method in prospective timing. The 11-point scale may reflect retrospective timing because it was conducted unexpectedly and postdictively, though participants knew that timing was a major task because of RP3. One of the purposes of this study was to reveal whether the return trip effect is observed when using prospective timing for a long real-life interval. The absence of the effect in RP3 indicates that the return trip effect is not induced in prospective timing. The difference between our results and Seno et al. [6] also using prospective timing could be attributed to duration intervals because their stimuli were 40 seconds. In addition to the two timing paradigms, Wearden [11] proposed another one, passage of time. Different from prospective and retrospective timings, which focus on how long a time period lasted, passage of time concerns how quickly time seemed to pass. By intuition, if time seems to pass quickly during an event, the event might be judged as short. However, Wearden [11] reported that film clip stimuli which seemed to pass more quickly were evaluated equally in retrospective time estimation. Passage of time may be easily influenced whereas retrospective timing appears to be difficult to manipulate. The return trip effect, which was only observed in the subjective scale judgment, may not be a matter of the duration judgment, but of passage of time. The question in the 11-point scale was "which movie they felt was longer" and the answer was for example "the first was a lot longer." This subjective scale may have been confused with passage of time. To investigate whether the 11-point scale was confused with passage of time, we should compare the postdictive verbal estimation and the 11-point scale with the same setting as the present experiment.

The absence of interaction in RP3 may reflect the specific timing method rather than the timing strategy. Previous studies have observed the return trip effect when using the verbal estimation and subjective scale methods [4,6], which suggests that the return trip effect can be assessed by both the method requiring quantification and that requiring just comparison [8]. As the production method used in RP3 seems homologous to the verbal estimation method [1], we had expected the return trip effect in RP3, but did not observe it. One of the possible differences between the production and the verbal estimation methods is a review of past time. In the production method, participants can predict how long they should pay attention to time because the target duration, 3 min in this study, is presented beforehand. On the contrary, in the verbal estimation method participants do not know how long they will measure time; presented durations may be 10 sec, 10 min, or 1 hour. In this unpredictable situation without counting strategies it may be more or less necessary to postdictively review past time after the task. The return trip effect observed in previous studies [4,6] using the verbal estimation may be attributed to the review of past time. The postdictive 11-point scale in the present study also includes the review of past time, which may imply the importance of the review of past time.

It is worth noting that the increase in RP3 in both the round-trip and the control condition in the second session might be attributable to repeated reports. According to interviews after completion of the tasks, participants initially seemed to find the RP3 task to be difficult, but as they continued they became accustomed to the task and found it easier. In general, the durations of simple or dull tasks tend to be underestimated, whereas complex or detailed tasks tend

to be overestimated [27–29]. Another possible explanation for the increase in RP3 is that there is a lag before the participant becomes absorbed by the movie. When playing a video game, players often underestimate the playtime, but when playing the game only briefly, they overestimate the playtime because of an "adaptation period" that is required to be fully immersed in the game [7,9,30]. Bisson et al. [9] discussed that the adaptation period might be less pleasant and thus induce overestimation of time. It is also possible to interpret the adaptation period from an attentional perspective: after this adaptation period, participants can be absorbed in the game, which distracts attention from its duration [12]. According to a model for apparent duration suggested by Glicksohn [31], apparent duration is a multiplicative function of the size of and the number of the subjective time unit. Externally oriented attention decreases the size of the time unit. In the present experiment, after an adaptation period in which participants might have been absorbed enough into the movie, more attention might have been deployed to the movie (an external stimulus) thereby decreasing the size of the time unit, and thus apparent duration might have been shortened. If the duration of the practice session is extended to fully elicit the possible habituation to the RP3 task or the possible absorption into a movie in advance, the change in RP3 observed in this study might vanish. Also, if absorption causes overproduction (underestimation) in RP3, RP3 while playing a video game could be increased after a certain adaptation period. These approaches could provide informative evidence about the change of RP3.

As mentioned above, the absence of the interaction in RP3 may reflect low statistical power. The difference between the two groups in the 11-point scale measure was very large, so the return trip effect seems to be prominently observed in postdictive time estimation. However, we can't be confident of the null results of RP3.

Moderately different influences on ANS

The two experimental conditions did not cause drastic, but only moderately different, changes in overall ANS activity. The whole ANS, as measured by SD-HR, was more active in the second trip session only for the control group. Similarly CSI, reflecting sympathetic activity, increased only for the control group. These results suggest that overall ANS activity differently responded for the two groups, mainly as a result of sympathetic activity. Increased HRV is associated with lower mental load [32,33]. However, the increase in sympathetic activity is considered to reflect an increase in mental stress or concentration [34]. It is difficult to infer the change in mental state in the control group. However, on the basis of the major contribution of sympathetic activity discussed above, it is possible that the control group might have felt greater mental stress in the second session. Watching one trip movie over 25 min was a lengthy task. During the second session participants in the control group might have felt that they would have to watch another long dull movie. In contrast participants in the round-trip group might have been relaxed because they would know it from past experiences of return trips that the return trip would seem short. Here we emphasize only that the combinations of movie-1 & -2 and movie-1 & -3 had moderately different influences on ANS activity.

Influence of ANS activity on time perception

Time perception, estimated postdictively, seems to be related to the parasympathetic activity. When the change in CVI between the two sessions was larger, participants in the control group felt that the session with larger CVI was shorter. The correlation between the change in SD-HR and the 11-point scale showed the same trend. On the basis of the fact that the parasympathetic nervous system activity represented by CVI contributes to ANS activity represented by SD-HR, and the values of r (.74 and. 62 for CVI and SD-HR, respectively), it can be inferred that

among ANS activities the parasympathetic activity mainly contributed to postdictive time perception. A recent study investigating the relationship between body signals and time perception suggests that the parasympathetic activity may affect time perception. In the reproduction method a decrease in HR caused by an increase of the parasympathetic activity during encoding of time improved the accuracy of duration reproduction [16]. In our study we cannot refer to the accuracy of postdictive time judgment, but the significant correlation between the 11-point scale and CVI may correspond to an improvement of the accuracy of time estimation. Participants may have tended to overestimate the duration, and the parasympathetic activity may have improved the accuracy of time estimation. As a result, the parasympathetic activity shortened time estimation and participants felt that the session with larger CVI was shorter. Contrary to the control group, the change in CVI was not related to the comparative judgment on the 11-point scale for the round-trip group. This indicates that the relationship between postdictive time perception and the parasympathetic activity may not be so robust.

At the end of this section we should represent one concern that the significant and nearly significant correlations out of 16 might well be false positives. In the present study CVI showed the significant correlation with time perception, which in part follows previous research [16] finding the relationship between time perception and the parasympathetic activity. Moreover, it seems reasonable that CVI and SD-HR showed higher *r* values because the parasympathetic nervous system composes ANS. So the correlations we found may be genuine. Nevertheless more research is need in this issue.

What causes the return trip effect?

Why does the round trip bias time perception? Ven et al. [4] reported that the return trip effect was observed not only when the return trip was via the route same as the initial trip, equivalent to our round-trip group, but also via a route different from the initial trip. Seno et al. [6] found that the return trip effect was induced only when self-motion perception was accompanied by a round-trip story. Though these two studies used shorter durations than the present study (7 min in Ven et al. [4], 40 s in Seno et al. [6]), their results both suggest that the fact or the awareness of "return" would be necessary for the return trip effect. Our control group did not have this awareness, which supports this idea. If this awareness is systematically manipulated, conditions necessary for the return trip effect might be found.

To interpret the return trip effect in a clearer way, the experimental design should be improved. The round-trip group watched movie-1 and -2 as a round trip while the control group saw movie-2 and -3 as a non-round trip. Due to this design the two groups were looking at different scenery or objects. We preliminarily searched the numbers of corners, distances, sizes of the roads, and traffic volume in order to match the environments of the routes. However, the scenery of the movies the two groups watched was not completely the same. For future research, one of the ways to solve this problem would be to use two sets of round-trip movies, such as pairs of movie-1 and -2, and movie-3 and -4. This design will enable us to make four round-trips and eight non-round-trips by counterbalancing the order and combination. Using this design, we will be able to compare the round-trip condition and the non-round-trip condition with the same scenery.

Also, the ecological validity could be elevated. We used as stimuli real-life intervals and the projection of walking movies, which seem to provide a sufficient sense of immersion. However, this environment is different from a real walk in some ways, for example, a narrow field of vision or the absence of physical activity. There are few studies relating time perception to physical activity, but physical activity may modulate time perception. It has been reported that physical activity lessened the accuracy and the variability of time perception [35]. In contrast,

Tobin & Grondin [8] found smaller variability of time perception with physical activity than visualizing that activity. The impact of walking as a physical activity should be investigated by using treadmill or a field study.

Conclusion

We investigated the return trip effect in a comparatively ecologically valid situation over a real-life interval. By comparing the round-trip condition and the non-round-trip condition, it was confirmed that the return trip does actually make us feel that time is shorter. Moreover, our two methods of time estimation suggest that the return trip effect does not affect the timing mechanism itself, but rather our feeling of time postdictively. We also examined whether ANS activity measured by ECG is related to time perception. Parasympathetic function is one of the resources for temporal information, although it is not so robust one.

For future research, it would be interesting to test the contribution of the awareness of "return" because this semantic labeling may be a major factor in inducing the cognitive bias of the return trip effect. Moreover, neuroimaging studies could provide insight into how time is perceived in ecological situations.

Author Contributions

Conceived and designed the experiments: RO KF MK. Performed the experiments: RO. Analyzed the data: RO KF MK. Contributed reagents/materials/analysis tools: RO KF MK. Wrote the paper: RO KF MK.

References

1. Coelho M, Ferreira JJ, Dias B, Sampaio C, Martins IP, Castro-Caldas A (2004) Assessment of time perception: the effect of aging. J Int Neuropsychol Soc 10: 332–341. PMID: 15147591
2. Buhusi CV, Meck WH (2005) What makes us tick? Functional and neural mechanisms of interval timing. Nat Rev Neurosci 6: 755–765. doi: 10.1038/nrn1764 PMID: 16163383
3. Meck WH (1996) Neuropharmacology of timing and time perception. Cogn Brain Res 3: 227–242. PMID: 8806025
4. Ven N, Rijswijk L, Roy MM (2011) The return trip effect: Why the return trip often seems to take less time. Psychon Bull Rev 18: 827–832. doi: 10.3758/s13423-011-0150-5 PMID: 21861201
5. Zakay D (2012) Experiencing time in daily life. Psychologist 25: 578–581.
6. Seno T, Ito H, Sunaga S (2011). Self-motion perception compresses time experienced in return travel. Perception 40: 497–499. doi: 10.1068/p6885 PMID: 21805925
7. Tobin S, Bisson N, Grondin S (2010) An ecological approach to prospective and retrospective timing of long durations: A study involving gamers. PLoS ONE 5: e9271. doi: 10.1371/journal.pone.0009271.t002 PMID: 20174648
8. Tobin S, Grondin S (2012) Time perception is enhanced by task duration knowledge: Evidence from experienced swimmers. Mem Cognit 40: 1339–1351. doi: 10.3758/s13421-012-0231-3 PMID: 22806428
9. Bisson N, Tobin S, Grondin S (2012) Prospective and Retrospective Time Estimates of Children: A Comparison Based on Ecological Tasks. PLoS ONE 7: e33049. doi: 10.1371/journal.pone.0033049.t002 PMID: 22412982
10. Wearden JH, Penton-Voak IS (1995) Feeling the heat: Body temperature and the rate of subjective time, revisited. Q J Exp Psychol 48: 129–141. doi: 10.1080/14640749508401443 PMID: 7597195
11. Wearden JH (2008) The perception of time: basic research and some potential links to the study of language. Lang Learn 58: 149–171.
12. Tobin S, Grondin S (2009) Video games and the perception of very long durations by adolescents. Comput Human Behav 25: 554–559. doi: 10.1016/j.chb.2008.12.002
13. Bar-Haim Y, Kerem A, Lamy D, Zakay D (2010) When time slows down: The influence of threat on time perception in anxiety. Cognit Emot 24: 255–263. doi: 10.1080/02699930903387603
14. Poynter WD (1983) Duration judgment and the segmentation of experience. Mem Cognit 11: 77–82. PMID: 6855562

15. Block RA (1982) Temporal judgments and contextual change. J Exp Psychol Learn Mem Cognit 8: 530–544. PMID: 6218220

16. Meissner K, Wittmann M (2011) Body signals, cardiac awareness, and the perception of time. Biol Psychol 86: 289–297. doi: 10.1016/j.biopsycho.2011.01.001 PMID: 21262314

17. Jamin T, Joulia F, Fontanari P, Giacomoni M, Bonnon M, Vidal F, et al. (2004) Apnea-induced changes in time estimation and its relation to bradycardia. Aviat Space Environ Med 75: 876–880. PMID: 15497368

18. Lediett V, Tong JE (1972) Effects of heart-rate increase on temporal discrimination and time judgment by two groups of delinquents. Percept Mot Skills 34: 759–764. PMID: 5040490

19. Smith A- L, Reynolds K (2006) Survey of Poincare indices for measuring heart rate variability. Australas Phys Eng Sci Med 29: 97–101. PMID: 16623229

20. Toichi M, Sugiura T, Murai T, Sengoku A (1997) A new method of assessing cardiac autonomic function and its comparison with spectral analysis and coefficient of variation of RR interval. J Auton Nerv Syst 62: 79–84. PMID: 9021653

21. Yamamoto Y, Hughson RL (1991) Coarse-graining spectral analysis: new method for studying heart rate variability. J Appl Physiol 71: 1143–1150. PMID: 1757311

22. Pagani M, Furlan R, Pizzinelli P, Crivellaro W, Cerutti S, Malliani A (1989) Spectral analysis of RR and arterial pressure variabilities to assess sympatho-vagal interaction during mental stress in humans. J Hypertens Suppl 7: S14–S15. PMID: 2632695

23. Malik M, Bigger JT, Camm AJ, Kleiger RE, Malliani A, Moss AJ, et al. (1996) Heart rate variability: Standards of measurement, physiological interpretation, and clinical use. Eur Heart J 17: 354–381. PMID: 8737210

24. Bos MG, Jentgens P, Beckers T, Kindt M (2013) Psychophysiological response patterns to affective film stimuli. PLoS ONE 8: e62661. doi: 10.1371/journal.pone.0062661 PMID: 23646134

25. Droit Volet S, Meck WH (2007) How emotions colour our perception of time. Trends Cogn Sci 11: 504–513. doi: 10.1016/j.tics.2007.09.008 PMID: 18023604

26. Faul F, Erdfelder E, Lang A-G, Buchner A (2007) G* Power 3: A flexible statistical power analysis program for the social, behavioral, and biomedical sciences. Behav Res Meth 39: 175–191. PMID: 17695343

27. Roy MM, Christenfeld NJS, Jones M (2013) Actors, observers, and the estimation of task duration. Q J Exp Psychol 66: 121–137. doi: 10.1080/17470218.2012.699973 PMID: 22928697

28. Roy MM, Mitten ST, Christenfeld NJS (2008) Correcting memory improves accuracy of predicted task duration. J Exp Psychol Appl 14: 266–275. doi: 10.1037/1076-898X.14.3.266 PMID: 18808280

29. Burt CD, Kemp S (1994) Construction of activity duration and time management potential. Appl Cogn Psychol 8: 155–168.

30. Rau P-LP, Peng S-Y, Yang C-C (2006) Time distortion for expert and novice online game players. Cyberpsychol Behav 9: 396–403. PMID: 16901242

31. Glicksohn J (2001) Temporal Cognition and the Phenomenology of Time: A Multiplicative Function for Apparent Duration. Conscious Cognit 10: 1–25. doi: 10.1006/ccog.2000.0468

32. Hill AB, Perkins RE (1985) Towards a model of boredom. Br J Psychol 76: 235–240. PMID: 4027489

33. Toichi M, Kubota Y, Murai T, Kamio Y, Sakihama M, Toriuchi T, et al. (1999) The influence of psychotic states on the autonomic nervous system in schizophrenia. Int J Psychophysiol 31: 147–154. PMID: 9987060

34. Kubota Y, Sato W, Toichi M, Murai T, Okada T, Hayashi A, et al. (2001) Frontal midline theta rhythm is correlated with cardiac autonomic activities during the performance of an attention demanding meditation procedure. Cogn Brain Res 11: 281–287. PMID: 11275489

35. Vercruyssen M, Hancock PA, Mihaly T (1989) Time estimation performance before, during, and following physical activity. J Hum Ergol 18: 169–179. PMID: 2637287
